# Artificial Professionalism: An Evaluation of Six Large Language Models on the UK Multi-Specialty Recruitment Assessment Professional Dilemmas Paper

**DOI:** 10.7759/cureus.115354

**Published:** 2026-08-28

**Authors:** Mohammad Jakir Ahmed, Munsif M Mansoor, Hamza A Mahmood, Rawan S Ebrahim

**Affiliations:** 1 General Internal Medicine, Watford General Hospital, London, GBR; 2 General Internal Medicine, Luton and Dunstable University Hospital NHS Foundation Trust, Luton, GBR; 3 General Internal Medicine, Frimley Health NHS Foundation Trust, Slough, GBR; 4 General Internal Medicine, Queen Elizabeth Hospital, London, GBR

**Keywords:** artificial intelligence, large language models, medical education, msra, professionalism, situational judgement test, specialty recruitment

## Abstract

Background: The Professional Dilemmas (PD) paper of the UK Multi-Specialty Recruitment Assessment (MSRA) is a situational judgement test (SJT) used to shortlist candidates for several postgraduate specialty training programmes, including general practice, core psychiatry, clinical radiology and obstetrics and gynaecology. Candidates increasingly use publicly available large language models (LLMs) informally during examination preparation, yet the performance of contemporary LLMs on this examination has not been established. This study aimed to quantify and compare the performance of six publicly available LLMs on the official MSRA practice paper.

Methods: The 22-item PD section of the NHS England (Workforce, Training and Education) 2023 MSRA official practice paper (maximum 86 marks) was administered in April 2026 to six publicly available LLMs via their free-tier consumer chat interfaces: ChatGPT (OpenAI), Microsoft Copilot, Claude (Anthropic), Gemini (Google), DeepSeek and Grok (xAI). Each item was entered verbatim with no system prompt. Responses were scored against the official answer key. Descriptive statistics, performance by item format, Spearman and Pearson inter-model correlations and a Friedman test for overall between-model differences were computed.

Results: All six models scored between 51/86 (59.30%) and 61/86 (70.93%), with a mean of 56.8/86 (66.09%; SD 4.06 percentage points). Gemini scored highest at 61/86 (70.93%) and Grok lowest at 51/86 (59.30%), but a Friedman test showed these differences were not statistically significant (χ²(5) = 7.64, p = 0.18). Across all models, mean performance on multiple-choice items at 26.5/33 (80.3%) markedly exceeded performance on ranking items at 30.3/53 (57.2%) (paired t = −16.1, p < 0.001). Models agreed completely on four of 22 items. Similarity in item-level performance patterns between models was modest (Spearman ρ 0.03-0.67).

Conclusions: Contemporary general-purpose LLMs achieve roughly two-thirds of available marks on the MSRA PD paper with no statistically significant differences detected between them, but reason far less reliably on ranking items than on multiple-choice items. Candidates should not treat any single LLM answer as authoritative and should refer to the primary sources on which the PD paper is built, such as the General Medical Council's Good Medical Practice.

## Introduction

The Multi-Specialty Recruitment Assessment (MSRA) is a computer-based examination used by NHS England and the devolved administrations to shortlist candidates for postgraduate medical training. A large proportion of UK resident doctors applying for specialty training each year sit the MSRA, and in several specialties, most notably general practice and core psychiatry, performance on the MSRA alone determines the offer of a training post [1]. The MSRA comprises a Clinical Problem Solving paper and a Professional Dilemmas (PD) paper. The PD paper is a situational judgement test (SJT) designed to assess the professional attributes expected of a Foundation Year 2 doctor, including probity, patient safety, coping with pressure and effective team-working [1].

SJTs were introduced into UK postgraduate medical selection in 2013, when an SJT became part of the UK Foundation Programme application process, following evidence from the occupational psychology literature that SJTs add incremental validity over academic measures alone in predicting professional behaviour [2-4]. Their subsequent adoption into specialty selection, including the MSRA, reflected a policy consensus that clinical knowledge is necessary but insufficient for safe practice, and that attributes such as integrity, resilience and teamworking are both assessable in structured selection formats [2] and predictive of subsequent workplace performance [3]. The MSRA PD paper now influences training offers across several UK specialty programmes, giving it a substantial and direct effect on the careers of UK-trained doctors. Crucially, the 2023 practice paper used in this study is the only publicly authorised MSRA question set released by NHS England, and is therefore the primary formative resource available to candidates.

Since the advent of large language models (LLMs), the volume of research evaluating their performance on medical examinations has grown substantially. ChatGPT has been shown to approach or exceed the pass mark of the United States Medical Licensing Examination and to perform comparably on UK undergraduate medical examinations [5,6]. Performance on non-clinical, judgement-based assessment is less well characterised. Borchert et al. reported that ChatGPT scored 76% on the 2023 UK Foundation Programme SJT practice paper but achieved full marks on only 9% of items, indicating that strong overall performance can coexist with inconsistent ethical reasoning [7]. A later study of the Association of American Medical Colleges SJT found that GPT-4 outperformed GPT-3.5 and Google Bard, but that all three models tended to make the same errors [8].

Candidates sitting the MSRA are predominantly Foundation Year doctors and doctors in non-training senior house officer-grade posts applying for entry into core and specialty training, rather than undergraduate medical students. Reported informal uses of LLMs in this context include generating additional practice scenarios once the single official practice paper has been exhausted, requesting explanations of why a particular ranking or selection is considered correct, rehearsing prioritisation and escalation reasoning against a model's suggested response and checking self-generated answers to the practice paper against a model's output. Each of these uses rests on an implicit assumption that the model's preferred answer approximates the official key.

Despite the widespread informal use of LLMs in MSRA preparation, no published data describe how these tools perform against the only officially sanctioned practice material. One plausible reason for this gap is that early reports suggest LLMs perform only modestly on judgement-based tasks relative to knowledge-based ones, which may have dampened academic interest. We, therefore, undertook a pilot benchmarking study of six publicly available LLMs on the NHS England 2023 MSRA practice paper, with the aims of (i) quantifying and comparing their performance, defined as raw marks awarded against the official answer key, and (ii) characterising the pattern and variability of their errors, defined as the dispersion of per-item scores within and between models and the similarity of item-level performance patterns across models.

## Materials and methods

**Figure 1:**
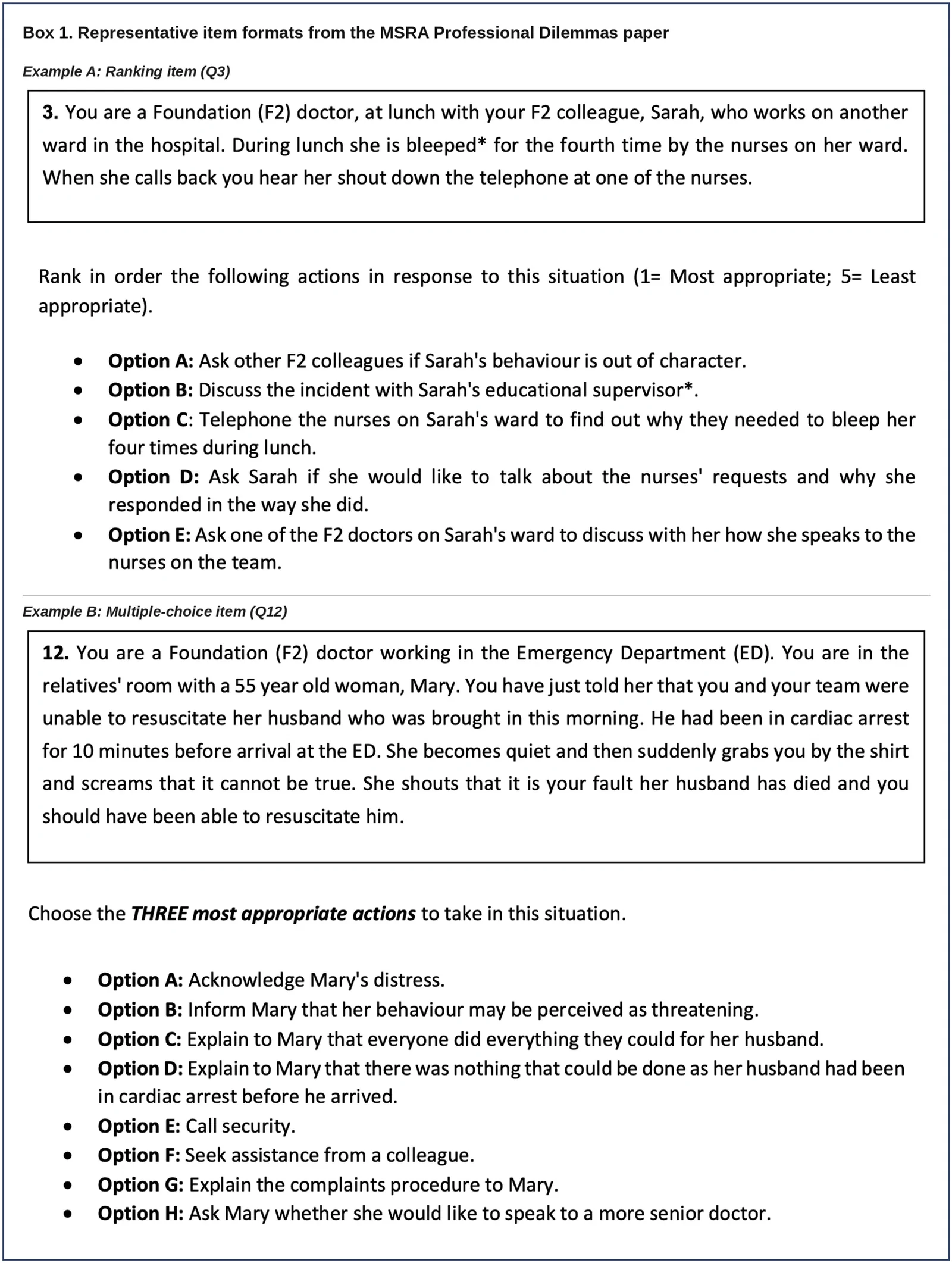
Representative item formats from the MSRA PD paper Example A: Item 3, a ranking item, in which candidates order five possible actions from most to least appropriate; Example B: Item 12, a multiple-choice item, in which candidates select the three most appropriate actions from eight options Items are reproduced verbatim from the NHS England 2023 MSRA practice paper [9]; contains public-sector information licensed under the Open Government Licence v3.0. MRSA: Multi-Specialty Recruitment Assessment; PD: Professional Dilemmas

The PD section of the official NHS England (Workforce, Training and Education; formerly Health Education England) MSRA practice paper (version 3, 2023) was used. This section contains 22 items, totalling 86 marks. The paper, its answer key and rationales are publicly available on the NHS England medical and dental recruitment website [9]. No negative marking is applied. Representative examples of each format are shown in Figure 1. Because the practice paper and its answer key have been publicly available online since 2023, the possibility that some or all models encountered these items, rationales or discussions of them during pre-training or via web-accessible content cannot be excluded; no test for memorisation or verbatim recall was performed.

Item formats

The section comprises two item formats. Ranking items present a clinical scenario followed by four or five possible actions; candidates order all options from most to least appropriate, with one mark awarded per option placed in the position specified by the official key. Multiple-choice items present a clinical scenario followed by eight or more response options; candidates select exactly three responses, with one mark per correct selection. Of the 22 items, 11 were ranking items (Q1-Q11; maximum 53 marks) and 11 were multiple-choice items (Q12-Q22; maximum 33 marks).

Models evaluated

Six general-purpose LLMs were evaluated on 24 April 2026 via their publicly available free-tier web chat interfaces, using each service's default model and settings with no paid features, model selection, or configuration changes: ChatGPT (GPT-5.3 Instant, OpenAI), Microsoft Copilot (Microsoft, underlying model not disclosed in interface), Claude (Claude Sonnet 4.6, Anthropic), Gemini (Gemini 3 Flash, Google), DeepSeek (DeepSeek-V3.2, DeepSeek) and Grok (Grok 4.1, xAI). Model versions are as documented in provider release notes and product documentation for the access date. Free-tier consumer access was used for every model, reflecting how candidates preparing for the MSRA are most likely to interact with these tools.

Procedure and scoring

Each item was administered once per model in a single run; no repeated administrations were performed. Each item was pasted verbatim into a fresh conversation, including the full scenario stem, response options and the official instruction. Each model's final answer (a ranked list or a set of three options) was recorded and compared against the official answer key, using the published marking scheme described above. Score concordance on an item was defined as complete when all six models returned an identical raw score for that item, irrespective of whether that score was correct.

Total raw scores and percentages were calculated. Descriptive statistics (mean, SD, range) were computed across models, and performance was compared by item format using a paired t-test and Wilcoxon signed-rank test. Pairwise inter-model similarity was assessed using both Spearman's rank correlation coefficient (ρ) and Pearson's r on per-item scores; given the ordinal nature of item scores, Spearman's ρ is treated as the primary measure. To test whether overall performance differed between models, a Friedman test was applied to per-item scores, with Kendall's W as the effect size. Statistical significance was set at α = 0.05. Analyses were performed in Python 3 (SciPy).

Ethical considerations

The study used only publicly available examination material and machine-generated responses; no human participant or patient data were involved. Formal ethical approval was therefore not required.

## Results

Overall performance

**Figure 2:**
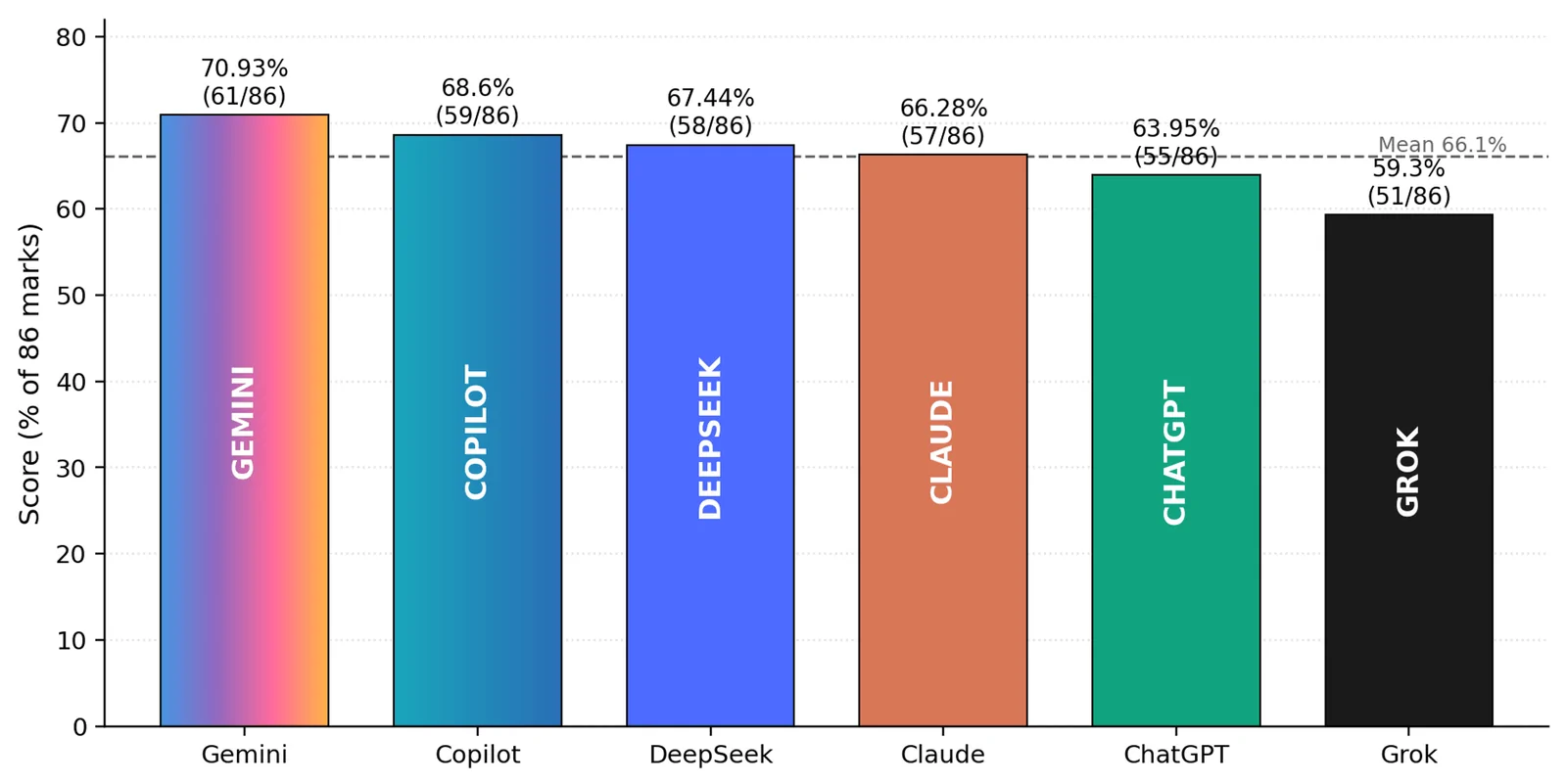
Overall performance of six LLMs on the MSRA PD paper Overall performance of six publicly available LLMs on the MSRA PD 2023 practice paper (22 items, 86 marks). The dashed line indicates the mean score across all models (66.1%). LLM: Large language model; MRSA: Multi-Specialty Recruitment Assessment; PD: Professional Dilemmas

All six models scored above 50% and below 75% of available marks. Gemini achieved the highest score (61/86, 70.93%), followed by Microsoft Copilot (59/86, 68.60%), DeepSeek (58/86, 67.44%), Claude (57/86, 66.28%), ChatGPT (55/86, 63.95%) and Grok (51/86, 59.30%). The mean score was 66.09% (SD 4.06 percentage points); the range between the highest- and lowest-scoring models was 11.63 percentage points, equivalent to 2.87 SDs. Overall results are summarised in Table 1 and Figure 2. However, a Friedman test across all 22 items showed that the differences between models were not statistically significant (χ²(5) = 7.64, p = 0.18; Kendall’s W = 0.07), indicating that the observed rank order should be interpreted with caution on this 22-item sample.

**Table 1:** Overall performance of six publicly available LLMs on the MSRA PD 2023 practice paper pp: Percentage points; LLM: Large language model; MRSA: Multi-Specialty Recruitment Assessment; PD: Professional Dilemma

Rank	Model	Raw score (/86)	Percentage	Deviation from mean (pp)
1	Gemini	61	70.93%	+4.84
2	Microsoft Copilot	59	68.60%	+2.51
3	DeepSeek	58	67.44%	+1.35
4	Claude	57	66.28%	+0.19
5	ChatGPT	55	63.95%	−2.14
6	Grok	51	59.30%	−6.79
—	Mean (SD)	56.8 (3.5)	66.09% (4.06%)	—

Performance by item format

**Figure 3:**
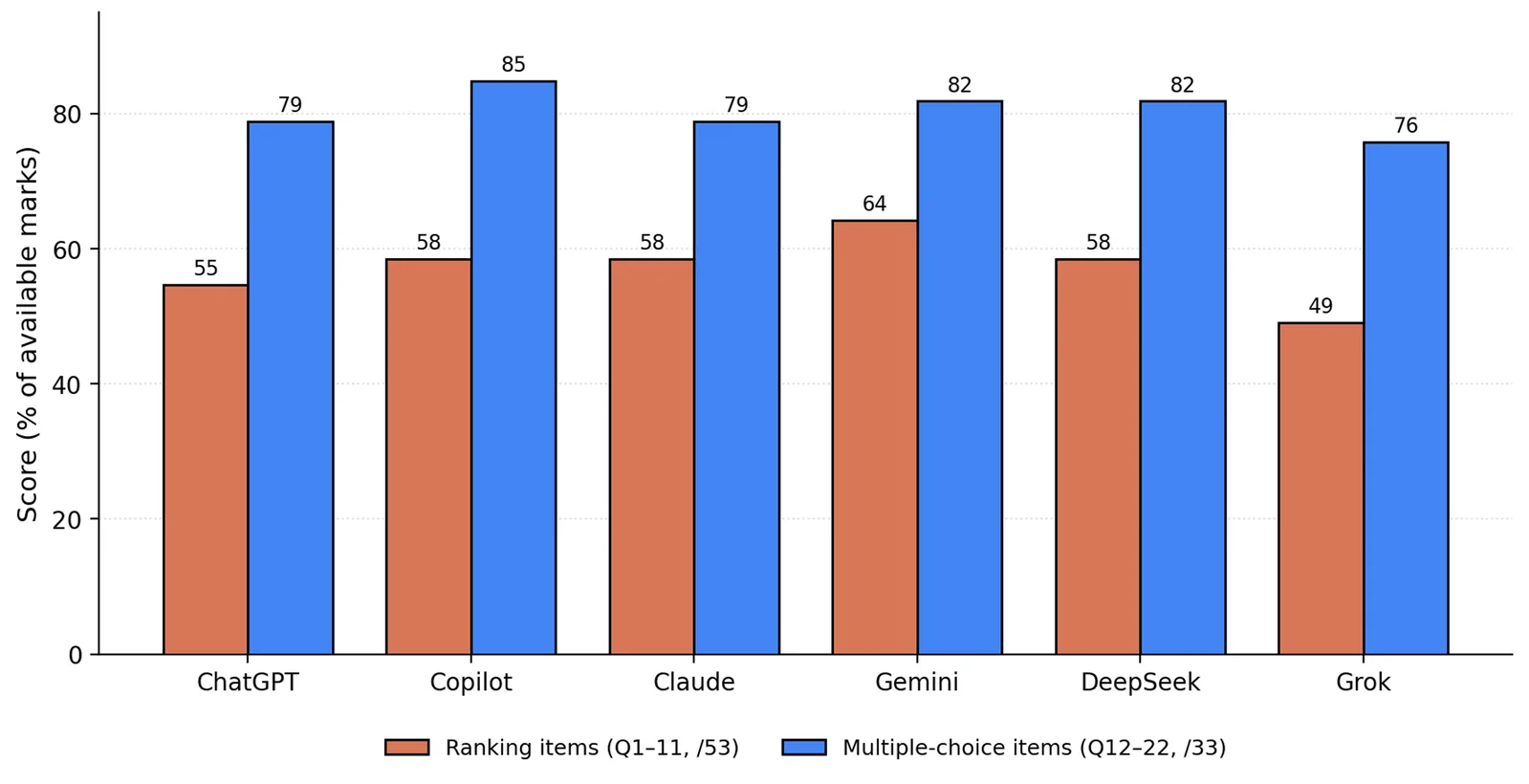
LLM performance disaggregated by item format: ranking vs multiple choice All models performed substantially better on multiple-choice items (select 3 of 8) than on ranking items (rank all 4–5 options), consistent with the marking scheme penalising near-misses in ranking items. LLM: Large language model

Performance differed markedly by item format (Figure 3). Across all six models, mean performance on multiple-choice items was 80.3% (SD 3.2) compared with 57.2% (SD 5.0) on ranking items, a difference of 23.1 percentage points that was consistent in direction across every model and statistically significant (paired t = −16.1, p < 0.001; Wilcoxon p = 0.031). No model scored below 25/33 (75.8%) on multiple-choice items, whereas the best ranking-item performance (Gemini, 34/53 (64.2%)) was lower than the worst multiple-choice performance (Grok, 25/33 (75.8%)). Per-model performance disaggregated by item format is shown in Table 2.

**Table 2:** Performance disaggregated by item format MCQ: Multiple-choice question; pp: Percentage points

Model	Ranking (Q1–11, /53)	MCQ (Q12–22, /33)	Difference (pp)
ChatGPT	54.70%	78.80%	24.1
Microsoft Copilot	58.50%	84.80%	26.3
Claude	58.50%	78.80%	20.3
Gemini	64.20%	81.80%	17.6
DeepSeek	58.50%	81.80%	23.3
Grok	49.10%	75.80%	26.7
Mean (SD)	57.2% (5.0)	80.3% (3.2)	23.1

Inter-model variability and similarity in performance patterns

Item-level variability was substantial. Score concordance was complete on four items (Q12, Q20, Q21, Q22), accounting for 12/86 (14.0%) of available marks; on none of these did the models achieve full marks. Conversely, the greatest dispersion occurred on Q8 (range 0-5, SD 1.72), Q4 (range 1-5, SD 1.55) and Q2 (range 0-4, SD 1.38), each a ranking item. On Q4 and Q8, a single model (Claude) scored full marks while others scored 1-2; on Q2, five models scored 0-2 while Grok scored 4. Gemini, Microsoft Copilot and DeepSeek were most often joint-highest scorers (15, 14 and 13 of 22 items, respectively), while Grok was joint-highest least often (9 items).

Pairwise correlations indicated only modest similarity in item-level performance patterns. Spearman's ρ ranged from 0.03 (Claude-Grok, p = 0.90, not significant) to 0.67 (ChatGPT-Gemini, p = 0.001); the corresponding Pearson values ranged from −0.23 (Claude-Grok; a negligible negative association, p = 0.30) to 0.63 (ChatGPT-Gemini, p = 0.002). The mean Spearman ρ across all 15 pairs was 0.40. Per-item raw scores for all models are provided in Table 3.

**Table 3:** Per-item raw scores for all six models Highest model(s) lists the model(s) achieving the top score on each item. Totals and overall percentages are shown in the final two rows. Q: Question number; Max: Maximum marks available for that item

Q	ChatGPT	Copilot	Claude	Gemini	DeepSeek	Grok	Max	Highest model(s)
1	4	2	2	4	4	4	4	ChatGPT, Gemini, DeepSeek, Grok
2	1	1	0	1	2	4	4	Grok
3	3	3	2	3	2	2	5	ChatGPT, Copilot, Gemini
4	1	1	5	2	2	1	5	Claude
5	5	5	3	5	2	3	5	ChatGPT, Copilot, Gemini
6	2	2	2	2	3	2	5	DeepSeek
7	3	5	3	3	3	3	5	Copilot
8	2	3	5	1	2	0	5	Claude
9	3	1	1	3	3	1	5	ChatGPT, Gemini, DeepSeek
10	2	5	5	5	5	3	5	Copilot, Claude, Gemini, DeepSeek
11	3	3	3	5	3	3	5	Gemini
12	2	2	2	2	2	2	3	ChatGPT, Copilot, Claude, Gemini, DeepSeek, Grok
13	1	2	2	2	2	2	3	Copilot, Claude, Gemini, DeepSeek, Grok
14	3	3	2	2	3	3	3	ChatGPT, Copilot, DeepSeek, Grok
15	3	3	3	3	3	2	3	ChatGPT, Copilot, Claude, Gemini, DeepSeek
16	1	2	1	2	2	1	3	Copilot, Gemini, DeepSeek
17	3	3	3	3	2	3	3	ChatGPT, Copilot, Claude, Gemini, Grok
18	2	2	2	3	2	2	3	Gemini
19	3	3	3	2	3	2	3	ChatGPT, Copilot, Claude, DeepSeek
20	3	3	3	3	3	3	3	ChatGPT, Copilot, Claude, Gemini, DeepSeek, Grok
21	3	3	3	3	3	3	3	ChatGPT, Copilot, Claude, Gemini, DeepSeek, Grok
22	2	2	2	2	2	2	3	ChatGPT, Copilot, Claude, Gemini, DeepSeek, Grok
Total	55	59	57	61	58	51	86	
%	63.95	68.6	66.28	70.93	67.44	59.3		

## Discussion

In this pilot study, six publicly available LLMs scored between 59% and 71% on the MSRA 2023 PD practice paper, with a mean of 66%. This range sits below the 76% reported by Borchert et al. for ChatGPT on the UK Foundation Programme SJT [7]. In a separate evaluation, Sareen assessed ChatGPT against SJT items from the Oxford Assess and Progress question bank and reported a score of 77.67% - again exceeding the performance observed here, although that question bank is a commercial revision resource rather than an officially sanctioned national assessment [10]. Several factors may explain the lower scores in our study. First, the PD paper targets Foundation Year 2 doctors and requires candidates to draw on lived NHS experience as well as understanding of probity, prioritisation and escalation. Second, the marking scheme we applied awards one mark per option in the exact correct position, penalising near-misses in ranking items more heavily than the partial-credit approach used operationally. Third, several models evaluated here were unavailable at the time of earlier studies, and some, notably Grok, may have had less targeted reinforcement on medical-professional content.

The format-dependent pattern observed here is consistent with broader literature indicating that LLM performance declines as tasks shift from factual recall towards judgement-weighted reasoning. On UK standardised admission tests, ChatGPT performed substantially worse on aptitude- and reasoning-focused examinations such as the Thinking Skills Assessment and Law National Aptitude Test than on knowledge-based science content [11]. Similarly, in medical ethics, ChatGPT achieved only moderate accuracy on bioethics question banks drawn from standard revision resources, with performance varying across ethical domains [12]. By contrast, GPT-4 scored at the 92nd percentile relative to applicant norms on the Association of American Medical Colleges PREview professional readiness examination [13]. A plausible explanation for this divergence is response format: PREview asks candidates to rate the effectiveness of each response independently, a task structurally closer to our multiple-choice items, whereas the MSRA ranking format requires a complete relative ordering, on which we found all six models weakest. This interpretation is strengthened by evidence that SJTs measure constructs distinct from cognitive ability and carry meaningful predictive validity for workplace performance [14], meaning that shortfalls against the official key cannot simply be dismissed as artefacts of an arbitrary marking scheme. Our finding that no significant between-model differences were detected also echoes recent comparative evaluations on medical multiple-choice questions (MCQs), in which leading chatbots including DeepSeek, ChatGPT and Gemini achieved broadly similar accuracy [15], suggesting that headline model choice may matter less than the nature of the task being asked of the model.

The most striking finding is the gap between item formats: every model performed far better on multiple-choice items (mean 80.3%) than on ranking items (mean 57.2%). This is consistent with the marking scheme, in which a single misplacement in a ranking item forfeits marks, whereas multiple-choice items reward identification of appropriate actions without requiring fine-grained ordering. It suggests that LLMs are reasonably able to recognise which actions are broadly appropriate but struggle to reproduce the precise prioritisation encoded in the official key. This mirrors the design intent of UK postgraduate selection, in which the SJT was introduced precisely because it assesses judgement distinct from the knowledge measured by clinical problem-solving tests [16]. For candidates, this implies that LLM output is least trustworthy precisely where the assessment is most demanding. 

Equally important, the apparent ranking of models was not statistically robust: the Friedman test did not detect a statistically significant difference between models; given only 22 items, this analysis was also limited in statistical power, so the absence of a detected difference should not be interpreted as evidence of equivalence. This reinforces that no single model can be treated as an authoritative answer key, because the models neither agree with the official key reliably nor differ meaningfully from one another. The modest inter-model correlations (mean Spearman ρ 0.40) further indicate that the models do not share a common profile of easy and hard items, weakening any case for using one LLM as a de facto key. The four items on which all models converged but none scored full marks suggest either a shared partial-credit attractor or a nuance in the official key that is under-represented in public training data; qualitative analysis of model rationales on such items is a logical next step.

Implications for medical education

This is, to our knowledge, the first study to benchmark multiple contemporary LLMs against the MSRA PD paper, the only publicly authorised mock for this component of UK specialty recruitment. Earlier benchmarking has focused on the UK Foundation Programme SJT or US licensing examinations; the MSRA PD paper has been overlooked despite its decisive role in specialty selection. The practice paper occupies a unique position as the sole MSRA-specific resource released for public use, and no prior study has used it to evaluate AI performance.

Anecdotal evidence indicates that many candidates already use general-purpose LLMs informally during preparation; querying model answers, generating scenarios or seeking explanations. This reflects a wider pattern of rapid, often unsupervised adoption of these tools across healthcare education, alongside documented concerns about accuracy and reliability [17]. A related development is the use of LLMs not only to answer practice material but also to generate it. Evaluations of AI-generated assessment content are mixed. LLM-generated examination questions derived from real-world electronic health records have been scored by blinded expert panels at levels approaching human-authored items overall, and higher than human authors on information correctness, although the corresponding AI-generated answers scored consistently below those written by clinicians [18]. In dermatology, AI-generated case scenarios were judged detailed in their descriptions but lacking the clinical depth, relevance and engagement of cases written without AI [19]. This matters directly for MSRA preparation, since informal and commercial question banks increasingly contain AI-crafted items: a model generating a PD scenario together with its own model answer would be operating precisely where our findings show its alignment with an official key to be weakest. Our data suggest this carries risk: a model scoring two-thirds of marks will endorse an incorrect answer or rationale in roughly one interaction in three, with no signal to the user that the response departs from the official key, and with reliability lowest on the ranking items that dominate the paper. It is also relevant that MSRA PD items are authored by practising NHS clinicians and psychometricians within a structured development framework and are calibrated against real candidate performance [20], with tests of this kind subject to formal validation both at admissions level and within individual specialties [21,22], whereas LLM outputs reflect public internet text.

One hypothesis is that the gap between model reasoning and the official key reflects a divergence in underlying value frameworks rather than simple factual error; however, the present quantitative data cannot distinguish this from alternative explanations, including differences in item interpretation, the exact-position scoring methodology, run-to-run model variability, or prior exposure to the questions. Qualitative analysis of model rationales would be required to adjudicate between these. LLMs may still hold educational value as tools for working through scenarios, provided users recognise that the model's preferred answer is an informed approximation rather than a normative one. Educators and resource providers should consider how the ready availability of moderate-quality LLM answers to published practice material affects the design of formative assessment banks, particularly given evidence that SJTs reliably capture non-academic attributes [23] and growing interest in using them not only for selection but also for teaching and assessing professionalism [24].

Limitations

Several limitations apply. First, MSRA results are reported operationally as scaled scores derived through equating and standardisation procedures that are not publicly available, and NHS England does not publish raw or scaled scores for the practice paper; the percentages reported here, therefore, have no published candidate distribution against which to benchmark. Publication of even summary statistics would permit direct human-AI comparison in future work. Second, the exact-position marking scheme makes the reported percentages a conservative estimate of equivalent candidate performance, since the operational scheme awards partial credit for near-correct positions. Third, only one attempt per model was performed. LLM outputs are stochastic rather than deterministic: identical prompts submitted on separate occasions can yield different responses, and repeated querying of the same medical examination items has been shown to produce inconsistent answers [25]. The scores reported here should therefore be understood as single-instance observations rather than stable estimates of model capability, and a repeat administration could plausibly produce a different rank order between models. A multi-run design capturing per-item response distributions would be required to quantify intra-model variance and to establish whether the ordering observed here is reproducible. Fourth, only 22 items were assessed, whereas the operational PD paper contains a larger and broader item set, which also limits the power of the between-model comparison. Fifth, the practice paper is publicly available and may be present in model training data, together with its official rationales and third-party discussion of the items; such contamination or memorisation could artificially inflate performance relative to unseen operational items. This is unavoidable in any study that, by regulation, must use non-live material.

## Conclusions

This pilot study demonstrates that contemporary, publicly available LLMs can engage credibly with the PD assessed in UK specialty recruitment, but that their answers align with the official key far less reliably when precise prioritisation is required than when broadly appropriate actions simply need to be identified. Because no model demonstrated clearly superior performance and all were individually fallible, no single model should be treated as a substitute for the official answer key or for authoritative professional guidance. For candidates preparing for the MSRA, LLMs are best regarded as scenario discussion partners whose preferred answers are informed approximations rather than normative ones.

Future work should establish whether these findings are stable across repeated runs, identify where model errors cluster by item format and professional domain, and examine how model reasoning aligns with UK professional standards. Publication of summary candidate performance data by the examination provider would enable direct comparison between human and model performance. Any future integration of these tools into formative preparation should be contingent on such evidence and developed collaboratively by clinicians, medical educators and developers.
